# Low radiation dose imaging of myocardial perfusion and coronary angiography with a hybrid PET/CT scanner

**DOI:** 10.1111/j.1475-097X.2008.00838.x

**Published:** 2009-01

**Authors:** S Kajander, H Ukkonen, H Sipilä, M Teräs, J Knuuti

**Affiliations:** 1 Turku PET CentreTurku, Finland; 2 Dept of Internal Medicine, Turku University HospitalTurku, Finland

**Keywords:** CT, CTA, hybrid imaging, PET, radiation dose

## Abstract

**Objectives:**

To test the image quality and feasibility of a sequential low radiation dose protocol for hybrid cardiac PET/CT angiography (CTA).

**Background:**

Multidetector computed tomography (MDCT) is a non-invasive method for coronary angiography. The negative predictive value of MDCT is high but perfusion imaging has a role in detecting functional significance of coronary lesions. This has encouraged combining these techniques. However, radiation dose is of concern. We report our first experiences with a low dose sequential CTA mode applicable to hybrid imaging.

**Methods:**

In the first phase, 10 consecutive cardiac MDCT angiographies were performed with spiral acquisition and compared in terms of image quality and dose with the following 10 patients performed with a new sequential mode. In the second phase, feasibility and radiation dose of a combined ^15^O-water rest-stress PET perfusion/sequential CTA protocol were assessed in another group of 61 consecutive patients.

**Results:**

Mean effective radiation dose was 60% lower in the sequential group than in the spiral group (19·3 versus 7·6 mSv, *P*<0·001). In the second phase, the new sequential hybrid protocol proved possible in 87% of the patients given the preconditions determined by the manufacturer. Mean effective dose of the CT acquisition was 7·6 mSv and total dose from the PET/CTA hybrid study 9·5 mSv.

**Conclusion:**

Low dose PET/CT allows cardiac hybrid studies with <10 mSv. The protocol can be applied to almost nine out of 10 patients with CT image quality comparable to spiral acquisition.

## Introduction

Until recently, evaluation of patients with suspected coronary artery disease (CAD) has been based on non-invasive methods of detecting myocardial ischemia followed by invasive coronary angiography. This paradigm, however, has recently been challenged by the development of coronary multi-detector CT (MDCT), which has emerged as an alternative for a non-invasive test of CAD. As an evolving method, its appropriate usage and indications are still evolving. Despite this, the number of coronary MDCT studies has increased rapidly as improvement in technology has occurred. The ability to non-invasively image coronary arteries, and to obtain important clinical information on the presence, severity, and characteristics of CAD including the visualization of luminal obstruction and atherosclerotic plaque, constitutes an attractive addition to previously available diagnostic tools (Berman *et al.*, 2006; Einstein *et al.*, 2007).

At present, a large number of studies on the diagnostic accuracy of MDCT have been published. In three recent meta-analyses, analysis of all coronary segments yielded segment-by segment sensitivity of 90–98% and specificity of 91–96% for 64 MDCT (Vanhoenacker *et al.*, 2007; Janne d′Othee *et al.*, 2008; Sun *et al.*, 2008). High negative predictive values (96–100% in segment-based analysis, 85–100% in patient-based analysis) suggest that 64 detector MDCT can reliably rule out the presence of hemodynamically significant (luminal narrowing >50%) CAD when compared against invasive angiography (De Feyter *et al.*, 2007; Sun *et al.*, 2008). Despite this, only moderate correlation between the severity of stenoses and myocardial perfusion abnormalities exists. A patient with normal perfusion may already have slight coronary changes that warrant proper medication (Schuijf *et al.*, 2006).

These findings suggest that CT angiography and myocardial perfusion imaging are complementary rather than substitutive methods to evaluate CAD (Schuijf & Bax, 2008). and that is why hybrid imaging such as cardiac PET/CT is likely to offer added value over CT only. The radiation dose of such combination, unfortunately, may be large. In addition, radiation due to a coronary CT angiogram on a 64 MDCT scanner is higher than with older 16 MDCT, and the dose received by the population is growing due to this technology and the increase of the studies performed (Berman *et al*., 2006; Hausleiter *et al.*, 2006; Einstein *et al.*, 2007; Nickoloff & Alderson, 2007; Paul & Abada, 2007).

The rationale of our study was to test the feasibility, image quality, diagnostic confidence and radiation dose of a low dose sequential (‘step-and-shoot’) cardiac CT protocol installed on a hybrid PET/CT scanner in a clinical setting. While some reports of this method performed on a stand-alone CT scanner are now available (Hsieh *et al.*, 2006; Earls *et al.*, 2008; Hirai *et al.*, 2008; Husmann *et al.*, 2008; Scheffel *et al.*, 2008), there are no studies in which this technique has been used as a part of a hybrid PET/CT study.

## Patients and methods

In the first part of the study, a standard spiral and a sequential, prospectively triggered MDCT protocol were compared by means of image quality and radiation dose. In all cases, the indication for the study was to rule out or to confirm suspected coronary artery disease and the field-of-view was determined accordingly. Patients with prior allergic reaction to iodinated contrast media or atrial fibrillation (FA) were excluded. After an initial acquisition to calculate calcium score, ten consecutive MDCT angiography studies were performed with spiral acquisition while the following ten patients were imaged using step-and-shoot mode if and when the inclusion criteria after possible premedication (sinus rhythm and HR <65 BPM with little variability) were met. The manufacturer set these suggested criteria.

In the second part, we tested whether the sequential imaging protocol is feasible in a larger series of patients. Sixty-two patients with suspected coronary artery disease, and who were scheduled for a hybrid cardiac PET/CT (and catheter angiography), were initially enrolled in this prospective study. Patients with FA were excluded again. One patient was omitted from the final analysis because MDCT acquisition failed due to injection pump malfunction. The final study population of this phase consisted of 61 patients.

**Figure 1:**
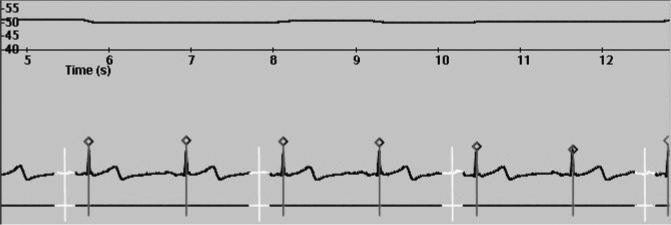
Principle of prospectively triggered step-and-shoot cardiac CT. Top: Heart rate trend graph; Bottom: Patient ECG during step-and-shoot CT angiography acquisition. White segments denote the time periods with X-ray tube on. White vertical line: 75% of the R-R-Interval. Vertical lines with diamonds: ECG triggering points.

All CT and PET scans were performed using a hybrid 64-row PET/CT scanner (GE Discovery VCT, General Electric, WI, USA). After initial lateral and frontal scout images, a preliminary non-enhanced data set were acquired to calculate the calcium score and to optimize the field of view in the CT angiography (CTA). 16 × 2·5 mm collimation, 120 kV tube voltage and 200 mAs tube current were used. The data in the contrast enhanced studies, both in sequential and spiral modes, were collected using the following parameters: 64 × 0·625 mm collimation, gantry rotation time of 350 ms, tube voltage of 120 kV (with the exception of one very small patient with whom 100 kV was used) and 600–750 mAs tube current depending on patient size. In the sequential mode, the pitch was always 1·0 with 5 mm overlap between slice stacks. Prospective ECG triggering determined the centre of the acquisition (end diastole, 75% of the cycle) while the width of this window was set between 100 and 200 ms according to HR (Fig. 1).

If spiral mode was used, the pitch was set between 0·16 and 0·24 according to HR. Routinely used ECG modulation software reduced radiation outside the 40–80% phase window by a maximum of 40%.

**Figure 2:**
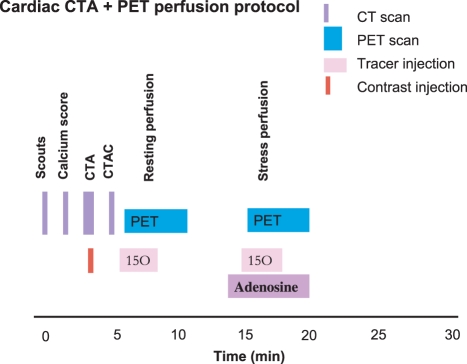
Combined cardiac CT angiography and PET perfusion protocol.

After CTA, rest-stress perfusion cardiac PET with ^15^O-water was performed as the second part of the one-stop-shop study. Cyclone 3 oxygen generator (IBA Molecular, Belgium) was used for ^15^O isotope production and ^15^O-water was produced with automatic Radio Water Generator (Hidex Oy, Finland). The combined PET/CT protocol is demonstrated in Fig. 2.

For CT analysis, all phases (0–90% in 10% intervals and the 75% phase) of the retrospectively gated spiral data sets were reconstructed retrospectively while all available data (usually 65–85% phases) of the sequential sets were reconstructed in 5% intervals. All data were sent to a GE ADW 4·4 workstation for analysis. In-house developed software (Carimas) was used to analyse ^15^O-water images after which we made PET/CT fusion images using CardiIQFusion software by GE.

The total radiation dose of the studies was recorded and the effective dose of the CT calculated with a method proposed by the European Working Group for Guidelines on Quality Criteria in CT (EC99 1999). The effective dose is derived from the product of the DLP and a conversion coefficient for the chest as the investigated anatomic region. This conversion coefficient (k = 0·017 mSv mGy^−1^ cm^−1^) is averaged between male and female models. ^15^O-water dose from two injections of 1100 MBq result to 2·05 mSv (Annals of the ICRP, 1998). The estimated dose from a similar study performed with ^82^rubidium, a more widely used alternative for measuring cardiac perfusion in PET, is reported as 13·5 mSv (Table 1).

**Table 1:** Typical radiation doses of diagnostic cardiac imaging modalities.

Method	Dose, mSv
^99m^Tc sestamibi rest-stress^a^	11·3
^201^Tl stress-reinjection^a^	31·4
^15^O-water^a^	2·5
^82^Rb^a^	13·5
^13^N-ammonia^a^	2·4
CTA (64 MDCT^a^)	8·4–21·4
Dual Source CT^b^	<10
Coronary Angiography^a^	2·3–22·7

a Einstein *et al.*, 2007.

b Achenbach *et al.*, 2008.

## Results

### Comparison of spiral and sequential CTA protocols

In the spiral group, eight of the 10 patients were female while in the sequential group there were six males and four females. The mean heart rates before the acquisition were 69·2 ± 9·7 and 62·2 ± 11·0 in the spiral and sequential groups, respectively (ns). Intravenous beta-blocker was administered in all patients in spiral group and in six patients in the sequential group (IV. metoprolol, 1 mg ml^−1^, 5–25 mg) to reach the target HR of <60. All 10 patients of the spiral group and eight of 10 in the sequential group were also administered isosorbide dinitrate about 5 min prior to the acquisition, the reason for not giving nitrate for the two being previously experienced headache due to similar drug in both cases.

No significant differences were present between the two patient populations in terms of patient size (body mass index, BMI) or HR (64·5 ± 14·1 versus 56·6 ± 5·9 BPM, *P* = 0·13) during the acquisition. The difference in mean HR in most part can be attributed to one single patient with a very high HR of 98. The patient characteristics are presented patient-by-patient in Table 2.

**Table 2:** Patient data and results of image quality, diagnostic confidence and radiation dose of spiral and sequential CTA (*n* = 20).

PT #	Gender	Protocol: 1 = SPIR, 2 = SEQ	BMI	HR Prior to acquisition	MG Metoprolol	Nitrate (Y/N)	HR During acquisition	Best ECG phase	Overall image quality 1–5 (1 = Poor, 5 = Excellent)	Artefact type	DG Confidence 1–5 (1 = Poor 5 = Excellent)	DLP (mGy*cm)	Calc.Eff.Dose (MsV)
1	F	1	20·4	63	25	Y	61	80	4	STAIR	5	866	14·7
2	F	1	22·4	80	25	Y	55	80	5	NONE	5	1064	18·1
3	F	1	25·7	65	10	Y	50	75	5	NONE	5	1076	18·3
4	F	1	28·3	64	10	Y	67	80LCA 40RCA	3	MOTION	4	1074	18·3
5	M	1	32·1	58	5	Y	63	80	3	NOISE	4	1369	23·3
6	F	1	43·0	75	20	Y	98	40	1	NOISE	2	1538	26·1
7	F	1	24·5	86	30	Y	77	40	3	MOTION	3	1053	17·9
8	F	1	20·4	59	5	Y	55	75	5	NONE	5	1005	17·1
9	F	1	28·7	64	15	Y	55	75	3	NOISE, STAIR	3	1364	23·2
10	M	1	23·7	78	25	Y	64	70LCA40RCA	4	NOISE	4	961	16·3
Mean ± SD			26·9 ± 6·8	69·2 ± 9·7	17 ± 9·2		64·5 ± 14·1		3·6 ± 1·3		4·0 ± 1·1	1137 ± 213	19·3 ± 3·6
11	M	2	27·6	68	15	N	61	75	4	NOISE	4	470	8·0
12	F	2	20·9	75	25	Y	61	75	4	STAIR	5	437	7·4
13	F	2	32·0	68	20	Y	61	75	3	NOISE	4	371	6·3
14	F	2	39·2	50	0	N	62	75	3	NOISE	4	495	8·4
15	M	2	26·9	50	0	Y	51	80	4	NONE	4	494	8·4
16	M	2	30·1	52	0	Y	52	75	4	STAIR	5	465	7·9
17	M	2	24·8	48	0	Y	44	80	5	NONE	5	491	8·3
18	F	2	24·1	64	15	Y	60	75	5	NONE	5	436	7·4
19	M	2	30·3	74	15	Y	56	75	2	NOISE, MOTION	3	472	8·0
20	M	2	31·1	73	30	Y	58	75	3	NOISE	4	366	6·2
Mean ± SD			28·7 ± 5·1	62·2 ± 11·0	12·0 ± 11·4		56·6 ± 5·9		3·7 ± 0·9		4·3 ± 0·7	450 ± 48	7·6 ± 0·8

The results of the assessments of image quality, reader confidence and radiation dose of the phase one patients are presented in Table 2. The studies were anonymized, blinded and mixed before an experienced radiologist evaluated them. Three separate readings were performed for each study and a mean whole number was recorded as the result. The studies were graded on a scale of 1–5 (with five being excellent and one poor) in terms of image quality and diagnostic confidence.

**Figure 3:**
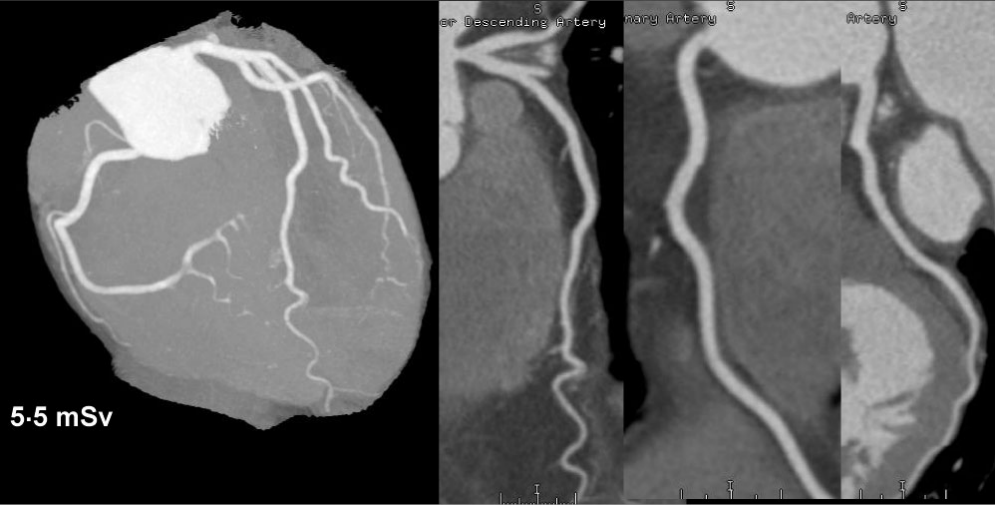
Example of sequential CT angiography with normal result. Radiation dose was 5.5 mSv.

There were no significant differences between the sequential and spiral groups in terms of either image quality or reader confidence. Mean image quality was 3·6/5 in the spiral group and 3·7/5 in the sequential group while the subjective diagnostic confidence of the reader was 4·0/5 and 4·3/5 in the same two groups, respectively (Fig. 3).

The mean effective dose was 19·3 ± 3·6 mSv in the spiral group (range, 15·0–26·0 mSv) and 7·6 ± 0·8 mSv (range, 6·0–8·4 mSv) in the sequential group. The mean dose reduction was 60·4% and the difference between the two groups statistically was significant (*P*< 0·001).

### The feasibility and radiation dose of sequential hybrid PET/CT protocol

In the second phase, the sequential technique proved feasible in 53 of the 61 patients (87%). The remaining eight patients (13%) had heart rates either too high (>65 BPM) or unstable even after maximum dose of i.v. metoprolol, or medication could not be given due to contraindications. These patients were studied with the spiral mode. Image quality between the spiral and sequential groups were not compared in this part of the study because of obvious preselection bias to each protocol. All studies, however, were of diagnostic quality.

In the second phase, all patients underwent a combined PET/CT study, of which the radiation doses are shown in Table 3. The mean total radiation dose from the sequential CT (calcium score and contrast enhanced CTA) was 7·6 ± 1·1 mSv (range, 5·9–10·2 mSv) whereas it was 19·9 ± 3·2 mSv in the eight patients that were scanned using spiral protocol. The mean radiation dose of the dual (rest-stress) ^15^O-water PET perfusion studies was 2·1 ± 0·1 mSv in the spiral patients yielding a total dose of 22·0 ± 3·2 mSv. Because the PET protocol remained unchanged regardless of the CT mode, the dose from PET was almost similar to the spiral group (mean, 2·0 ± 0·2 mSv). The mean total dose of the combined study was 9·5 ± 1·5 mSv with sequential CT acquisition. This analysis included 50 patients because PET failed in three subjects due to a technical failure in the ^15^O-water system.

**Table 3:** Radiation dose (mSv) of spiral and sequential hybrid CT/PET.

Scan Type	CTA + calcium score	^15^O-water PET	combined protocol
Spiral (*n*= 8)	19·9 ± 3·2	2·1 ± 0·1	22·0 ± 3·2
Sequential (*n* = 50)	7·6 ± 1·1	2·0 ± 0·2	9·5 ± 1·5
*n =* 58	*P <*0·001	*P <*ns	*P <* 0·001

**Figure 4:**
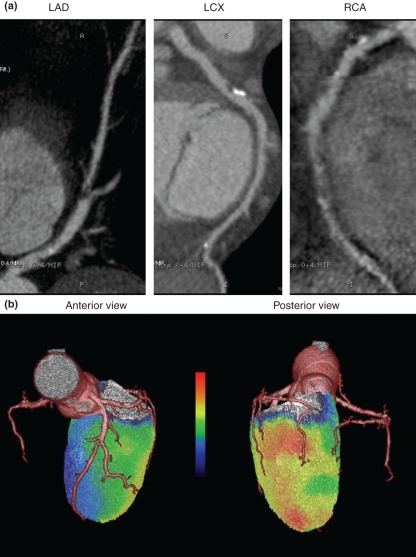
Example of hybrid PET perfusion and CT angiography. a: CT angiography (spiral mode) shows multi-vessel disease with occluded left anterior descending artery (LAD) and poorly visualized mid right coronary artery (RCA). Moderate disease in left circumflex artery LCX. b: Hybrid display during adenosine stress demonstrates severe perfusion reduction in LAD associated region (anterior view) while perfusion was best preserved in LCX related region (posterior view). Perfusion was colour scaled so that red colour denotes 3.5 ml/min/g. Resting perfusion was normal (not shown). Total radiation dose of the hybrid study was 22.3 mSv.

**Figure 5:**
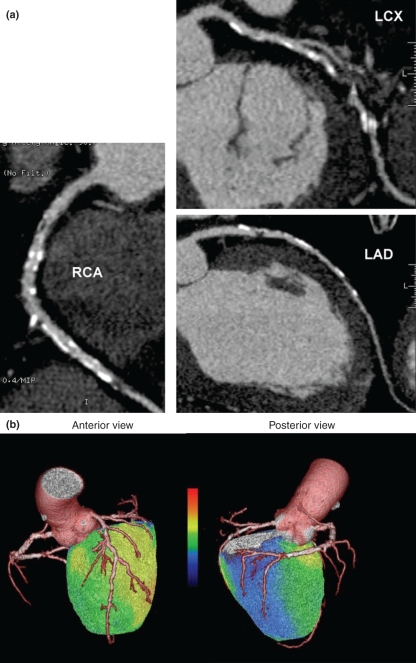
Example of hybrid PET perfusion and CT angiography. a: CT angiography (step-and-shoot mode) shows multi-vessel disease with massive coronary calcifications and severe stenoses in all major coronary arteries. b: Hybrid display during adenosine stress demonstrates quite preserved perfusion in anterolateral wall but moderate reduction in septal wall (anterior view). In RCA related region the perfusion was the most severely reduced (posterior view) indicating culprit stenosis in this vessel. Perfusion was colour scaled so that red colour denotes to 3.5 ml/min/g. Resting perfusion was normal (not shown). Total radiation dose of the hybrid study w as 11.3 mSv.

Examples of two hybrid-imaging cases and their corresponding doses are presented in Figs 4 and 5.

## Discussion

The results of the present study show that a comprehensive non-invasive imaging of both coronary angiography and rest-stress myocardial perfusion can be performed with radiation dose <10 mSv in nine out of 10 patients. The results also suggest that both the image quality and clinical confidence of MDCT are preserved.

Radiation dose is becoming a major issue for cardiac imaging. Even before the current CT-driven growth, the volume of cardiac diagnostic procedures involving the use of ionizing radiation – both within nuclear and invasive cardiology – increased considerably. Due to improved accuracy, the number of cardiac CT scans is growing rapidly and is expected to increase (Berman *et al.*, 2006; Einstein *et al.*, 2007). The mean radiation dose of 64 MDCT cardiac studies has recently been estimated as 15·2 mSv for males and 21·4 mSv for females (De Feyter *et al.*, 2007). Skin, breast, oesophagus and heart have the highest recorded absorbed organ doses. Of particular concern is the female breast that may receive a dose 10–30 times larger than received by mammography screening (Nickoloff & Alderson, 2007). In fact, the relative risk for breast cancer incidence for girls and women is estimated to be 1·004–1·042 for a single examination (Hurwitz *et al.*, 2007). In comparison, diagnostic invasive selective coronary angiography has a mean effective radiation dose of 2·3–22·7 mSv, and nuclear perfusion imaging with SPECT has a mean effective radiation dose of ∼15–20 mSv (Einstein *et al.*, 2007).

The primary ways to avoid unnecessary radiation are, of course, proper patient selection and preparation. Although data from high-probability patients is still scarce, it seems that CTA is at its best in low to medium risk groups. Massive coronary calcifications deteriorate image quality and it has been stipulated that patients with very high calcium scores should be omitted from CTA in favour of other methods. Patient preparation should be optimized including adequate heart rate control if necessary.

Classic methods to decrease dose by reducing tube current and voltage and increasing the pitch are widely used but always compromise image quality at least to a certain degree. Anatomy based current modulation depends on tissue attenuation; the more attenuating tissue there is in the field of view the more radiation is used. An even more sophisticated method, ECG-dependent X-ray tube current modulation, reduces current only at phases outside the critical time window between end systole and end diastole (Kalender *et al.*, 1999; Jung *et al.*, 2003; Morin *et al.*, 2003; Prinak *et al.*, 2006; Fei *et al.*, 2008).

The most recent innovations in CT dose containment largely depend on new technical advances and the need of new hardware such as twin tube systems or wide 256- or even 320-slice detector arrays. It is likely that these machines do improve image quality to dose ratio especially at higher heart rates (McCollough *et al.*, 2007; Achenbach *et al.*, 2008; Rybicki *et al.*, 2008; Stolzmann *et al.*, 2008).

Our approach, sequential step-and-shoot imaging with prospective ECG-triggering, utilizes existing hardware and is essentially a modification of traditional axial imaging adapted to a wide MDCT detector. The advantage of this technique is the relative ease of implementation: it can be used with existing scanners including some hybrid PET/CT models. Although an early report indicates that it may offer diagnostic CTA at doses as low as 1–2 mSv (Husmann *et al.*, 2008), we chose to use the mode with a wider time window to ensure uncompromised image quality. Even so, the technique reduced dose by more than 60% as compared to spiral imaging with similar current, slice thickness and voltage.

In addition to not to be able to scan patients with high or unstable heart rates, there appears to be just a single main limitation using the sequential technique, namely the lack of anatomical information from phases outside the predetermined time window. In contrast to ECG-modulated spiral imaging, the step-and-shoot technique does not provide images of every phase, thus disabling evaluating cardiac motion and functional measures such as ejection fraction.

PET imaging of myocardial perfusion is a method to obtain complementary functional information and also to quantify absolute tissue perfusion when a suitable tracer is employed. When a short-lived radioisotope (such as ^15^O-water) is used, a complete functional study to diagnose possible CAD may be performed in a single session with a very low radiation dose. In select cases, CT may also serve as a gate keeper as the first part of a one-stop-shop protocol: a normal CTA effectively rules out significant CAD (‘no coronary artery disease present’) while even a patient with normal perfusion – if haemodynamically non-significant coronary artery plaques are seen at CT- may benefit from proper medication. Therefore, in our opinion, a hybrid protocol that combines low dose CT with low dose PET is desirable.

The principle limitation of the first part of our study is the lack of anatomical gold standard such as catheter angiography to confirm the good, although subjective image quality based on the findings of a single reader. The good accuracy of step-and-shoot technique has been, however, evaluated in a recent paper (Scheffel *et al.*, 2008). The performance of spiral 64 coronary MDCT is now generally well established.

Statistically non-significant difference between average heart rates between the two groups may have had effect on image quality. Another major, although in our opinion unavoidable limitation is that the reader cannot work completely blinded to the used protocol due to obvious signs in images such as more visible change in contrast media concentration between the image stacks in sequential data sets.

Although reports indicate that females receive higher effective doses from coronary CT than males, we did not select patients on the base of their gender. This may have exaggerated the difference of dose between the groups in the first phase. On basis of the literature, however, it is unlikely that this difference is large enough to explain but a minor fraction of the reduction.

## Conclusion

Sequential cardiac low dose CTA is feasible and can be implemented into existing CT and hybrid PET/CT hardware. It lowers CT dose by 60% with comparable image quality and can be performed on almost nine out of ten patients. When combined with a low-dose PET perfusion protocol, a complete one-stop-shop cardiac exam with both anatomical and functional information may be obtained at <10 mSv.
